# Towards nano-optical tweezers with graphene plasmons: Numerical investigation of trapping 10-nm particles with mid-infrared light

**DOI:** 10.1038/srep38086

**Published:** 2016-12-01

**Authors:** Jianfa Zhang, Wenbin Liu, Zhihong Zhu, Xiaodong Yuan, Shiqiao Qin

**Affiliations:** 1 College of Optoelectronic Science and Engineering, National University of Defense Technology, Changsha 410073, China; 2 State Key Laboratory of High Performance Computing, National University of Defense Technology, Changsha, 410073, China

## Abstract

Graphene plasmons are rapidly emerging as a versatile platform for manipulating light at the deep subwavelength scale. Here we show numerically that strong optical near-field forces can be generated under the illumination of mid-IR light when dielectric nanoparticles are located in the vicinity of a nanostructured graphene film. These near-field forces are attributed to the excitation of the graphene’s plasmonic mode. The optical forces can generate an efficient optical trapping potential for a 10-nm-diameter dielectric particle when the light intensity is only about about 4.4 *mW*/*μm*^2^ and provide possibilities for a new type of plasmonic nano-tweezers. Graphene plasmonic tweezers can be potentially exploited for optical manipulation of nanometric biomolecules and particles. Moreover, the optical trapping/tweezing can be combined with biosensing and provide a versatile platform for studing biology and chemistry with mid-IR light.

Plasmonics provide a powerful platform for manipulating light-matter interactions. Recently, graphene plasmons have attracted much attention. The low-loss intrinsic plasmons in graphene exhibit small spatial extensions and remarkable enhancement of local electromagnetic fields^1^,^2^. Along with the tunability, graphene plasmons are rapidly emerging as a versatile platform for manipulating light at the deep subwavelength scale^3^,^4^. The exploration of graphene plasmonics has lead to the proposition and demonstration of a variety of functionalities in mid-infrared and THz ranges such as graphene waveguides^5^,^6^, absorbers^7^,^8^,^9^, photodetectors^10^,^11^, tunable metamaterials^12^,^13^, filters and polarizers^14^,^15^, and others^16^,^17^.

Optical forces play an important role for light-matter interactions at the mesoscale. In visible and near-infrared ranges, strong light-driven near-field forces in various metallic plasmonic structures have been studied^18^,^19^,^20^. And the ability of metallic nanostructures to control light at the subwavelength scale have been exploited to design plasmonic nano-optical tweezers which can realize optical trapping down to the nanometre scale^21^,^22^,^23^. Very recently, optical gradient forces enhanced by graphene plasmons have been investigated^24^,^25^,^26^. Here we study the optical gradient forces in graphene plasmonic structures and exploit it for nano-optical trapping in the mid-infrared range. We show numerically that strong optical near-field forces can be generated under the illumination of mid-IR light when dielectric nanoparticles are located in the vicinity of a nanostructured graphene film. This near-field force is linked to the excitation of the graphene’s plasmonic mode. The optical forces can generate an efficient optical trapping potential for a 10-nm-diameter dielectric particle.

## Results and Discussion

**Figure 1:**
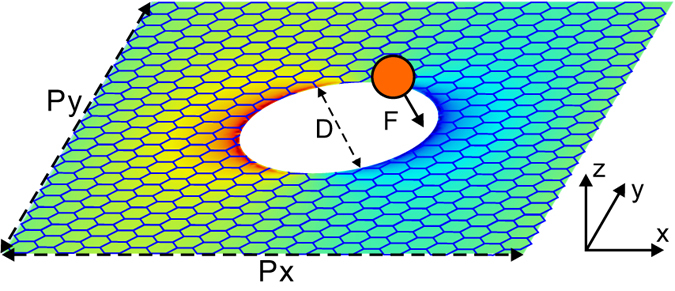
Trapping nanoparticles with graphene plasmonic structures. This figure shows the schematic of the proposed system along with geometric parameters. The graphene film is patterned with a periodical array of circular holes. The period is *Px* = *Py* = 400 nm and diameter of the hole is *D* = 150 nm. The diameter of the dielectric nanoparticles is 10 nm.

We investigate a periodical system which could be employed for the parallel trapping of nanoparticle arrays. Figure 1 shows the schematic of the system to be studied. We assume that periodical array of nanoparticles are located near circular nanoapertures in a doped graphene film. The period of the graphene nanoaperture array is *Px* = *Py* = 400 nm and the diameter of circular apertures is *D* = 150 nm. The diameter of the nanoparticles is *s* = 10 nm and the particles are assumed to be lossless with a refractive index of 2.0. We assume the Fermi energy of graphene to be *E*_*F*_ = 0.6 eV which corresponds to a doping density of about 2.6 × 10^13^ *cm*^−2^ and may be realized by chemical or electrostatic doping^27^.

**Figure 2:**
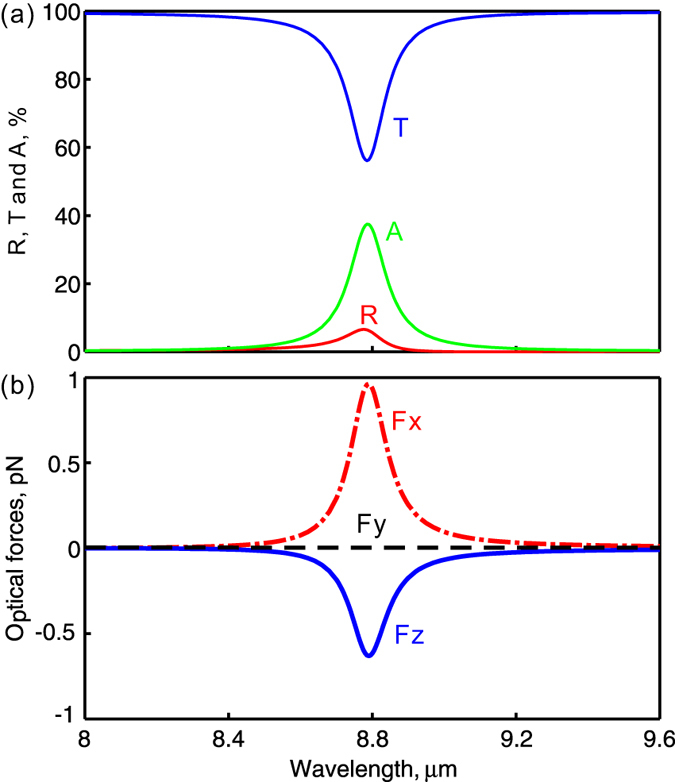
Plasmonic resonances and giant gradient optical forces. (**a**) Simulated reflection, transmission and absorption spectra with the illumination of mid-infrared plane wave at normal incidence; (**b**) Calculated optical forces exerted on the nanoparticle. The Fermi energy of graphene is *E*_*F*_ = 0.6 eV.

Figure 2a shows the simulated reflection, transmission and absorption spectra. Here the center of the nanoparticle is located 10 nm above the graphene film and is 60 nm away from the center of the hole in the x-direction. A plane wave with its electric field polarized along x-direction is illuminated on the graphene film at normal incidence. There is a strong resonance at around *λ* = 8.8 *μm* due to the excitation of localized plasmons in the graphene film. The localized plasmons lead to the light trapping, local field enhancement and strong optical field gradients around the circular apertures. Figure 2b shows the optical forces exerted on the dielectric nanoparticle. Forces are evaluated using the Maxwell stress tensor integral formalism (see Methods). Strong resonant optical forces are exerted on the dielectric nanoparticle around the plasmonic resonance. The x-direction component of the optical force is positive. Meanwhile, the z-direction component is negative and it pulls the nanoparticle towards to the graphene film. At the same time, the y-component of the force is zero when the particle locates at the *y* = 0 nm position due to symmetry.

**Figure 3:**
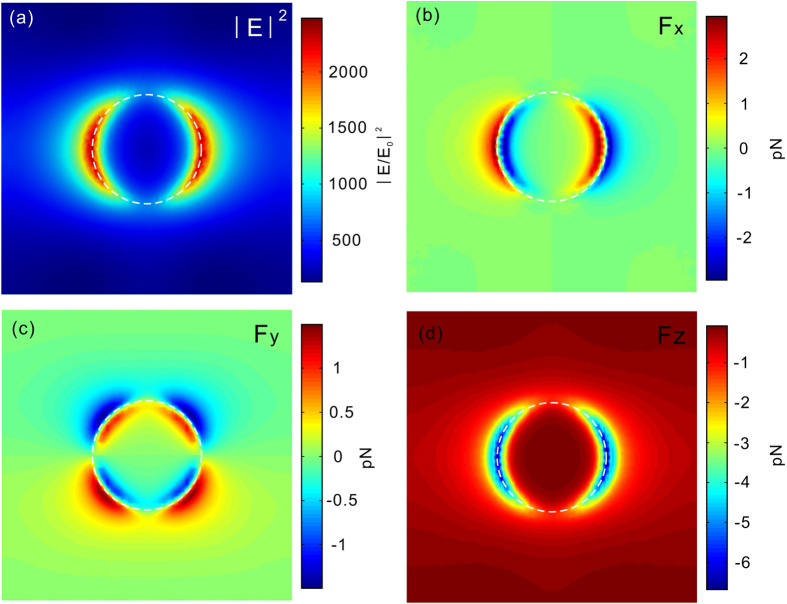
(**a**) Normalized electric field intensities at the resonance wavelength of *λ* = 8.8 *μm*. The field distribution is plotted at 10 nm above the graphene (*z* = 10 nm) and normalized to the electric intensity of incident light; (**b**,**c**) The transverse components of optical forces exerted on a 10-nm-diameter dielectric nanoparticle; (**d**) The normal component of the optical force. The refractive index of the particle is *n* = 2 and it is placed 10 nm above the graphene. The incident light is x-polarized and its optical intensity is assumed to be 10 *mW*/*μm*^2^. The Fermi energy of graphene is *E*_*F*_ = 0.6 eV.

Figure 3a shows the field distributions in the x-y plane (*z* = 10 nm) at the resonance wavelength of *λ* = 8.8 *μm*. Most of the field is located around the left and right sides of the circular aperture where the intensity of the near field varies dramatically with positions. Figure 3b and c display the transverse components of the optical forces (x- and y-components respectively). The optical force is linearly proportional to the intensity of incident light. Here we assume that the intensity of the incident light is *I* = 10 *mW*/*μm*^2^. The two transverse components of the optical forces work to trap the nanoparticle around the hot spots of the electromagnetic field. When the nanoparticle locates at the centre of the hot spots where the intensity of electric field is maximum, the transverse components of the optical forces are zero in both x- and y-directions. If the nanoparticle moves away from the centre of the electromagnetic hot spots, the transverse components of the optical forces will pull it back to the centre of the hot spots. Meanwhile, the nanoparticle also experiences a normal optical force component (*F*_*z*_) which pulls the particle toward the circular aperture (Fig. 3d). Here the normal component is much stronger than the transverse components of the optical force and can be used to confine the nanoparticle to the graphene surface.

**Figure 4:**
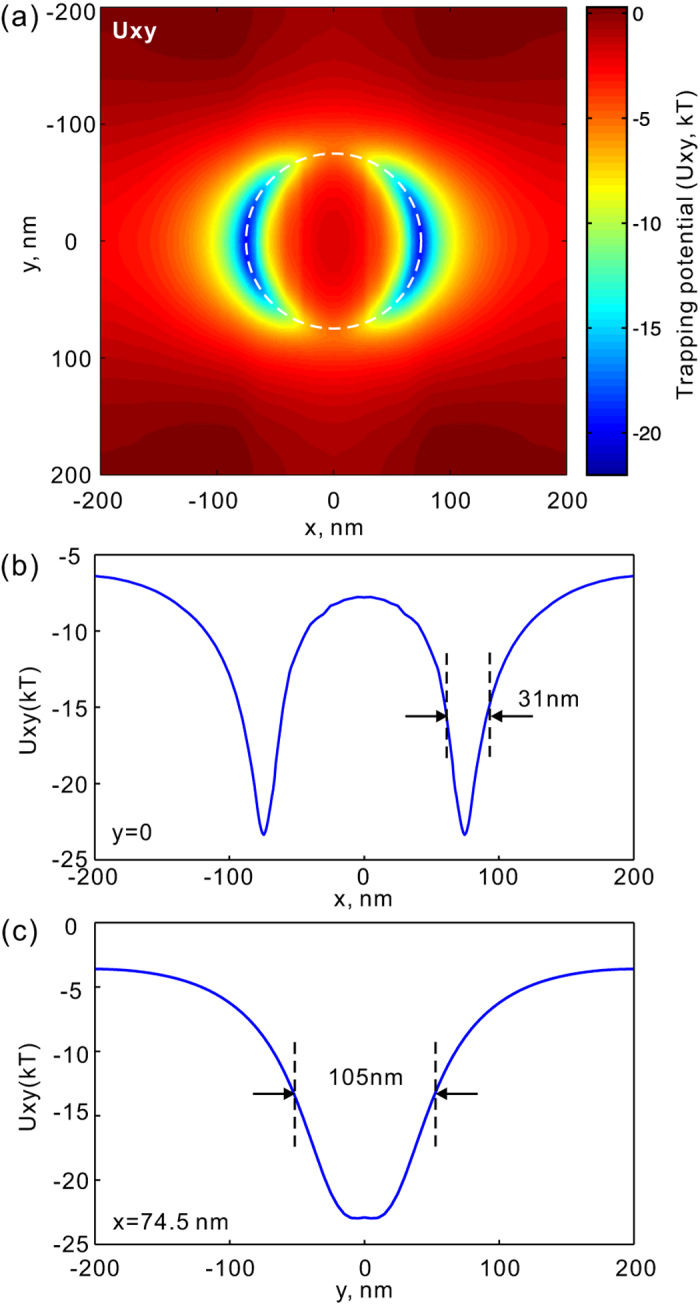
(**a**) Transverse optical trapping potential generated by the transverse optical forces (*F*_*x*_ and *F*_*y*_) on a 10 nm dielectric particle; (**b**,**c**) The variation of transverse optical trapping potential (*U*_*xy*_) along *y* = 0 nm and *x* = 74.5 nm. The full-width at haft maximum (FWHM) of the trapping potential is also indicated.

Figure 4a shows transverse trapping potential produced by the transverse optical force components in the x-y plane (*z* = 10 nm) at the resonance wavelength of *λ* = 8.8 *μm*. The trapping potential determines the stability of the optical trap and it is directly obtained from the restoring optical forces through an integration. It defines an important figure of merit for an optical trap. A dual trapping potential well is generated. The depth of the well is about 23 kT when the intensity of incident light is *I* = 10 *mW*/*μm*^2^. Here *k* is Boltzmann’s constant, and *T* = 300 K is the temperature. We consider 10 kT as the threshold for stable optical trapping^28^, so trapping this 10 nm dielectric particle requires an intensity of about 4.4 *mW*/*μm*^2^. Due to the stronger confinement of electromagnetic field along the x-direction, the optical trap is tighter in the x-direction (*FWHM* = 31 nm) compared to the y-direction (*FWHM* = 105 nm), as shown in Fig. 4b and 4c. The trapping efficiency here is comparable to that of optical traps realized by metallic plasmonic structures in the visible and near infared ranges^29^.

**Figure 5:**
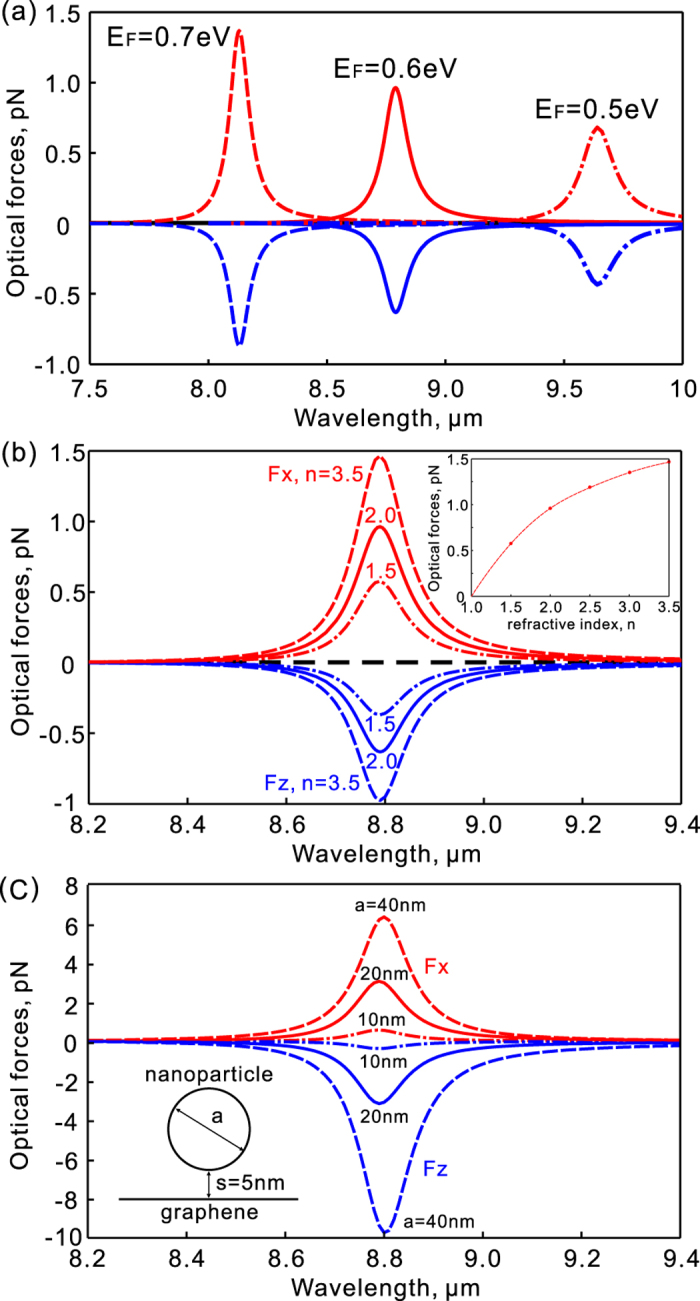
Optical forces exerted on the nanoparticle with the variation of parameters. (**a**)Variation of the Fermi energy of graphene. The diameter of the nanoparticle is 10 nm and its refractive index is n = 2. (**b**) Variation of the nanoparticle’s refractive index. The diameter of the nanoparticle is 10 nm and the Fermi energy is *E*_*F*_ = 0.6 eV. (**c**)Variation of the particle size. The refractive index of nanoparticle is n = 1.5 and the distance between the bottom of the particle and the graphene film is fixed to *s* = 5 nm as shown in the inset. Other parameters are the same as in Fig. 2.

One of the most important properties of graphene plasmons is the tunability. Plasmonic resonances of graphene nanostructures can be controlled by changing the Fermi energy through electrostatic gating. As shown in Fig. 5a, the plasmonic resonances shifts to shorter wavelengthes with the increase of the Fermi energy. Meanwhile, the plasmonic resonance become stronger and the maximum resonant forces exerted on nanoparticle increase. This additional tunability can hardly be realized in metal plasmonic tweezers and may be explored for manipulation with new freedoms. Another important parameter that affects the performance of trapping is the refractive index of the trapped nanoparticle. As shown in Fig. 5b, the optical force in the x-direction increases from about 0.58 pN to about 1.46 pN when the refractive index increases from 1.5 to 3.5. So it is relatively easier to trap nanoparticles with higher refractive indices (see the inset of Fig. 5b). Figure 5c shows the optical forces for nanoparticles with different sizes. Here the distance between the bottom of the nanoparticle and the graphene film is fixed to be 5 nm (see the inset of Fig. 5c) and the refractive index is 1.5. The optical force in the x-direction increases from 0.58 pN to about 3.1 pN when the diameter of the nanoparticle changes from 10 nm to 20 nm. It further increases to about 6.3 pN when the diameter of the nanoparticle increases to 40 nm. The force increases faster at the beginning because the electromagnetic field is confined in the deep subwavelength area around the graphene nanohole.

Optical trapping have been well studied in the past decades^30^ and various methods have been developed for optical nano-trapping such as metal plasmonic tweezers^29^,^21^,^31^, slot waveguide^32^, photonic crystal resonator^33^ and tapered optical fiber^34^,^35^. For the proposed graphene plasmonic nano-trapping, the required light intensity for efficient trapping is comparable to that of metal plasmonic trapping^31^ as well as other conventional methods^30^. It shows remarkable trapping capability for nanoparticles down to ten nanometers or even smaller with nanoscale precision which is comparable to metal plasmonic tweezers and can outperform other trapping methods including the nano-trapping methods with photonic crystal resonators or tapered optical fiber. Another potential advantage of the proposed graphene plasmonic nano-trapping is the tunability of graphene plasmons through electrostatic gating which may provide additional freedoms for manipulating nanoparticles. Most importantly, it works with mid-IR light which can be combined with infrared spectroscopy and shows great potentials for chemical and biological applications. For practical applications, heating effect is an important issue to be considered in plasmonic trapping. As graphene shows very good thermal conductivity and here the required optical intensity is comparable to conventional and plasmonic tweezers, we believe that the heating problem can be solved with suitable designs.

## Conclusions

In summary, we show numerically that strong optical near-field forces can be generated on a 10-nm-diameter dielectric nanoparticle when it is located in the vicinity of a nanostructured graphene film and illuminated by mid-IR light. This type of near-field forces are attributed to the excitation of graphene plasmons. Due to the local field enhancements and small spatial extensions, graphene plasmons produce large field gradients in the deep sub-wavelength area around the resonance frequency and thus strong optical gradient forces. Efficient optical trapping can be realized for a 10 nm nanoparticle whose diameter is three orders smaller than the wavelength of trapping light with an intensity of only a few *mW*/*μm*^2^. Indeed, it is also possible to trap particles with larger sizes (e.g., for a 60 nm-diameter dielectric particle with a refractive index of 2.0, the generated optical forces on it are one order stronger and up to tens of pN when the center of the nanoparticle is located 50 nm above the graphene film and is 60 nm away from the center of the hole in the x-direction) as well as sub-10 nm particles. This provides possibilities for a new type of plasmonic nano-tweezers. As a demonstration, we have only discussed graphene film with periodical nanostructures where an array of nanoparticles can be trapped simultaneously. It is also possible to trap nanoparticles with a single hole or other nanostructures in a graphene film. Moreover, we can tune the properties of graphene plasmons by varying the doping level and thus provide additional control over the trapping. Graphene plasmonic tweezers can be potentially exploited for optical manipulation of nanometric biomolecules and particles. Moreover, the optical trapping/tweezing can be combined with biosensing^36^ and provide a versatile platform for studying biology and chemistry with mid-IR light.

## Methods

The numerical simulations are conducted using a fully three-dimensional finite element technique (in Comsol MultiPhysics). In the simulation, the graphene is modelled as a conductive surface^5^,^37^. The sheet optical conductivity of graphene can be derived within the random-phase approximation (RPA) in the local limit^38^


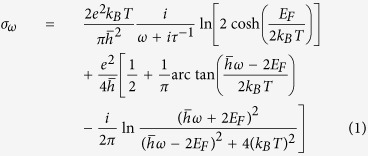


Here *k*_*B*_ is the Boltzmann constant, *T* is the temperature, *ω* is the frequency of light, *τ* is the carrier relaxation lifetime, and *E*_*F*_ is the Fermi energy. *E*_*F*_ depends on the concentration of charged doping and 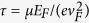, where *v*_*F*_ ≈ 1 × 10^6^ *m*/*s* is the Fermi velocity and *μ* is the dc mobility. Here we use a moderate mobility *μ* = 10000 *cm*^2^ · *V*^−1^ · *s*^−1^. And the temperature is assumed to be *T* = 300 *K*. The first term in Eq. (1) corresponds to intra-band transitions and the second term is attributed to inter-band transitions.

Forces are evaluated using the Maxwell stress tensor. Within the framework of classical electrodynamics, the total time-averaged electromagnetic force **F** exerted on a nanoparticle illuminated with light can be calculated using a surface integral:


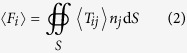


where *S* is a closed surface that enclosed the particle and *T*_*ij*_ is the time-averaged Maxwell stress tensor:


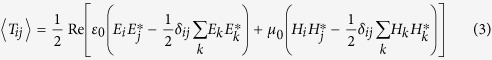


The stress tensor integral equation (2) encompasses both the radiation pressure and near-field optical gradient force. Here the radiation pressure on the dielectric nanoparticle is negligible compared to the optical gradient force.

## Additional Information

**How to cite this article**: Zhang, J. *et al*. Towards nano-optical tweezers with graphene plasmons: Numerical investigation of trapping 10-nm particles with mid-infrared light. *Sci. Rep.* **6**, 38086; doi: 10.1038/srep38086 (2016).

**Publisher's note:** Springer Nature remains neutral with regard to jurisdictional claims in published maps and institutional affiliations.
